# Author Correction: Genetic architecture of host proteins involved in SARS-CoV-2 infection

**DOI:** 10.1038/s41467-021-21370-6

**Published:** 2021-02-02

**Authors:** Maik Pietzner, Eleanor Wheeler, Julia Carrasco-Zanini, Johannes Raffler, Nicola D. Kerrison, Erin Oerton, Victoria P. W. Auyeung, Jian’an Luan, Chris Finan, Juan P. Casas, Rachel Ostroff, Steve A. Williams, Gabi Kastenmüller, Markus Ralser, Eric R. Gamazon, Nicholas J. Wareham, Aroon D. Hingorani, Claudia Langenberg

**Affiliations:** 1 MRC Epidemiology Unit, University of Cambridge, Cambridge, UK; 2 Institute of Computational Biology, Helmholtz Zentrum München – German Research Center for Environmental Health, Neuherberg, Germany; 3 Institute of Cardiovascular Science, Faculty of Population Health Sciences, University College London, London, WC1E 6BT UK; 4 UCL BHF Research Accelerator centre, London, UK; 5 Department of Medicine, Brigham and Women’s Hospital, Harvard Medical School, Boston, MA USA; 6 Massachusetts Veterans Epidemiology Research and Information Center (MAVERIC), VA Boston Healthcare System, Boston, Massachusetts USA; 7 SomaLogic, Inc., Boulder, CO USA; 8 The Molecular Biology of Metabolism Laboratory, The Francis Crick Institute, London, UK; 9 Department of Biochemistry, Charité University Medicine, Berlin, Germany; 10 Vanderbilt Genetics Institute, Vanderbilt University Medical Center, Nashville, TN USA; 11 Health Data Research UK, Wellcome Genome Campus and University of Cambridge, Cambridge, UK; 12 Health Data Research UK, Institute of Health Informatics, University College London, London, UK; 13 Computational Medicine, Berlin Institute of Health (BIH), Charité University Medicine, Berlin, Germany

**Keywords:** Genetics, Biomarkers

Correction to: *Nature Communications* 10.1038/s41467-020-19996-z, published online 16 December 2020.

The original version of this Article cited “Mehra, M. R., Desai, S. S., Kuy, S., Henry, T. D. & Patel, A. N. Cardiovascular disease, drug therapy, and mortality in Covid-19. *N. Engl. J. Med*. **382,** e102 (2020)” as Ref. 20. The cited paper was retracted; accordingly, Ref. 20 has been replaced with "Grasselli G et al. Risk factors associated with mortality among patients with COVID-19 in intensive care units in Lombardy, Italy. *JAMA Intern. Med.* **180**, 1345–1355 (2020)”. This has been corrected in the PDF and HTML versions of the article.

