# Supplementary material for: Genetic architecture of host proteins involved in SARS-CoV-2 infection
Source: Nat Commun. 2020 Dec 16;11:6397. doi: 10.1038/s41467-020-19996-z (PMC7744536; doi:10.1038/s41467-020-19996-z)
Supplement: Supplementary file 1 — Supplementary Information [file 41467_2020_19996_MOESM1_ESM.pdf]

Supplementary Information

Genetic architecture of host proteins involved in SARS-CoV-2 infection

Maik Pietzner[1], Eleanor Wheeler[1], Julia Carrasco-Zanini[1], Johannes Raffler[2], Nicola D. Kerrison[1], Erin Oerton[1], Victoria P.W. Auyeung[1], Jian'an Luan[1], Chris Finan[3,4], Juan P. Casas[5,6], Rachel Ostroff[7], Steve A. Williams[7], Gabi Kastenmüller[2], Markus Ralser[8,9], Eric R. Gamazon[1,10], Nicholas J. Wareham[1,11], Aroon D. Hingorani[3,4,12*], Claudia Langenberg[1,8,11,13*]

FIGURES

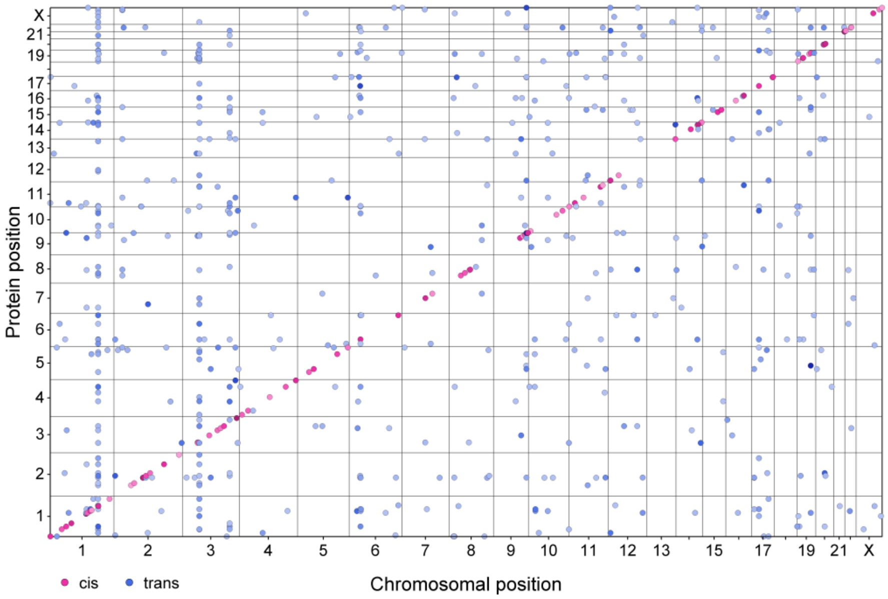

Figure S1 Genomic location (x-axis) of single nucleotide polymorphisms (SNPs) identified to be significantly associated ($p<5x10^{-8}$) with at least one of the 179 COVID-19 relevant proteins (y-axis, sorted by genomic location of the protein encoding gene). Pink circles indicate SNPs in close proximity to the protein encoding gene (±500kb, *cis*-protein quantitative trait loci) and blue circles those elsewhere in the genome.

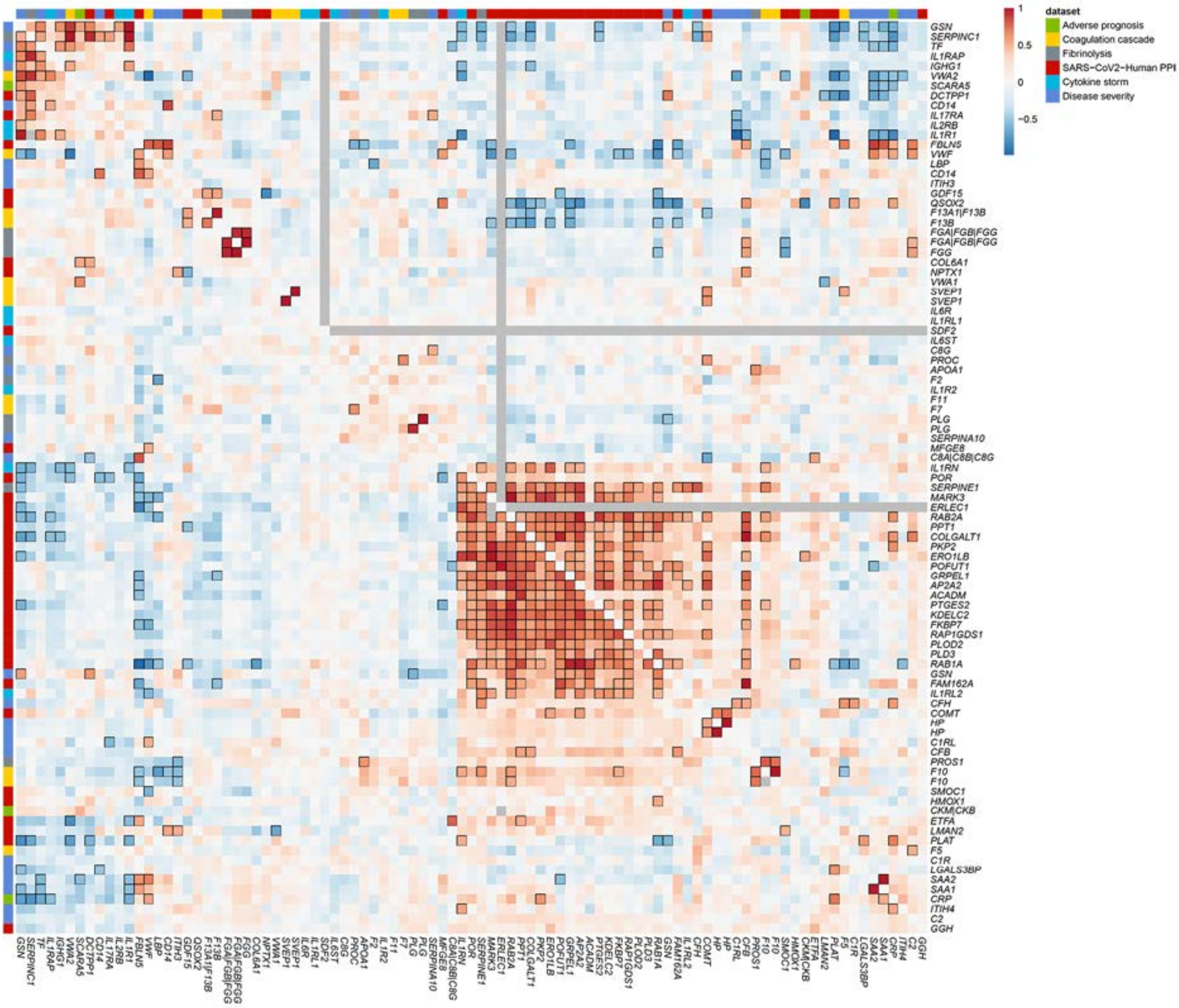

Figure S2 Genetic correlation of 93 Somamers regressing out the top sentinel cis-pQTL (lower triangle) and excluding pleiotropic trans genomic regions (upper triangle). SOMAmers were clustered based on absolute genetic correlations and protein encoding genes were used as labels. The column on the far left indicates relevance to SARS-CoV2 infection. Strong correlations (|r|>0.5) are indicated by black frames. Values for proteins with low heritability estimates (heritability < 1.5 x SE(heritability)) in these sensitivity analyses are shown in grey.

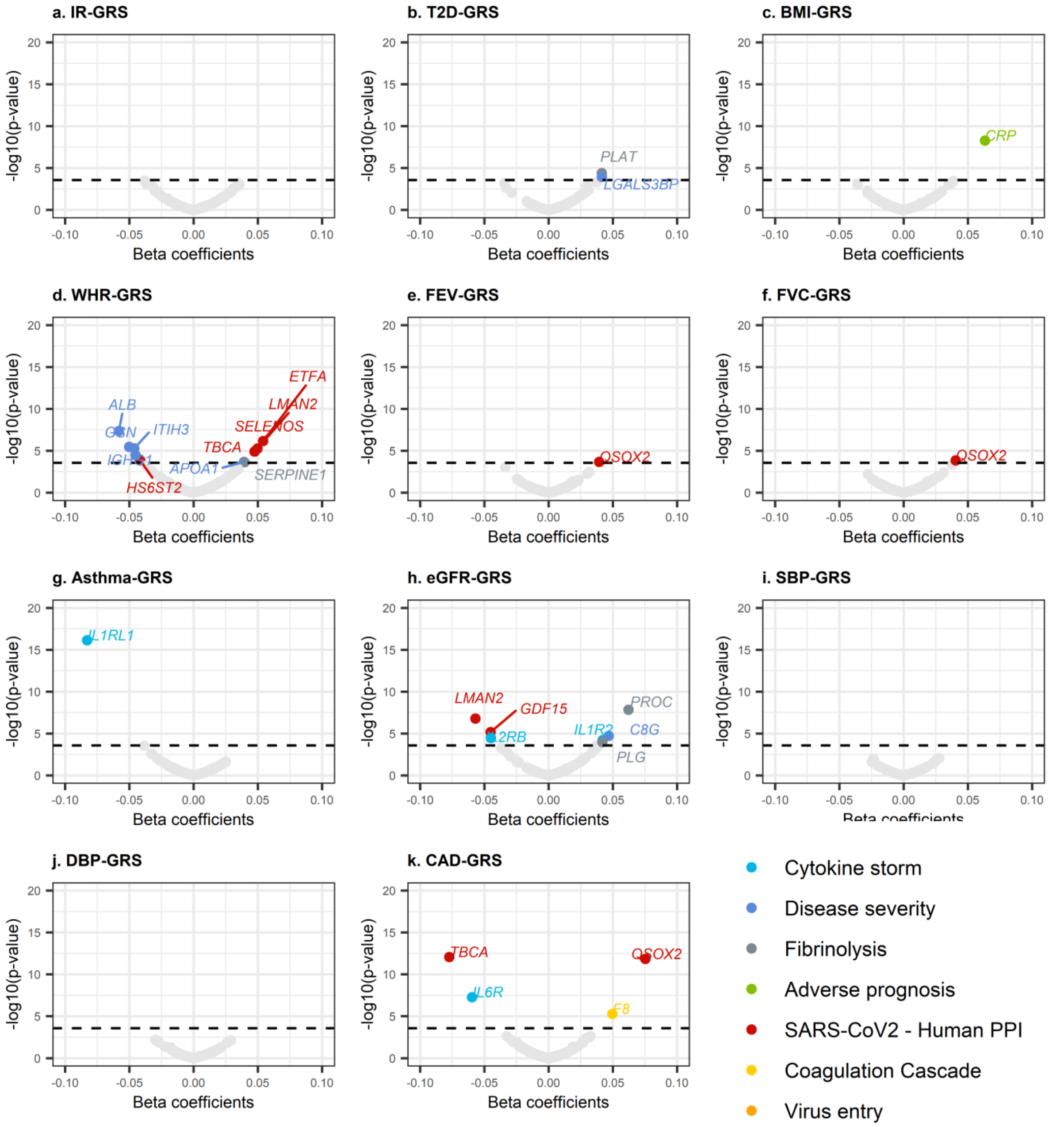

Figure S3 Differences in plasma abundances of 186 Somamers targeting candidate proteins by genetic susceptibility to diseases and risk phenotypes. Each panel shows the association of genetic risk scores for metabolic (T2D = type 2 diabetes, IR = insulin resistance, BMI = body mass index, WHR = waist-to-hip ratio), respiratory (FEV = forced expired volume, FVC = forced vital capacity, Asthma) and cardiovascular (eGFR = estimated glomerular filtration rate, SBP = systolic blood pressure, DBP = diastolic blood pressure, CAD = coronary artery disease) phenotypes with protein levels. Significant associations (p-value < 0.05/186 for multiple comparisons) are coloured according to COVID-19 associated processes.

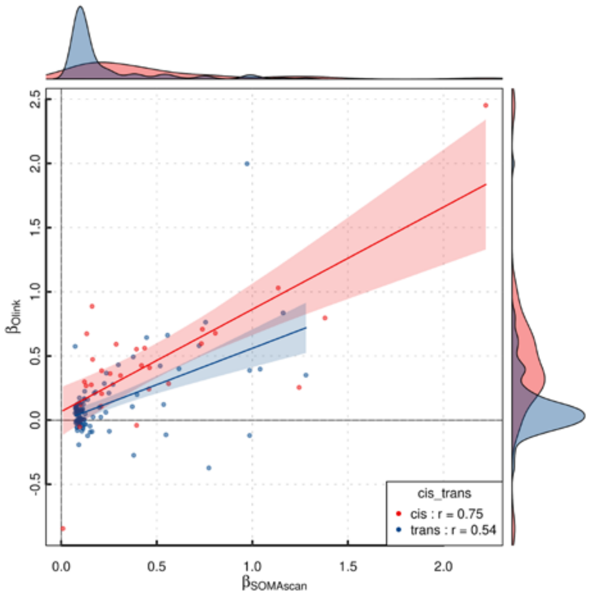

Figure S4 Comparison of effect estimates for protein quantitative loci (pQTL) discovered using either the SOMAscan or Olink platforms for 32 proteins targeted by both platforms. Colours indicate the proximity to the protein encoding gene (red - *cis*, blue - *trans*). Correlation coefficients (r) are given stratified by cis/trans status. Effect estimates have been aligned to be positive for all aptamer associations to enable unbiased assessment of the correlation coefficient.

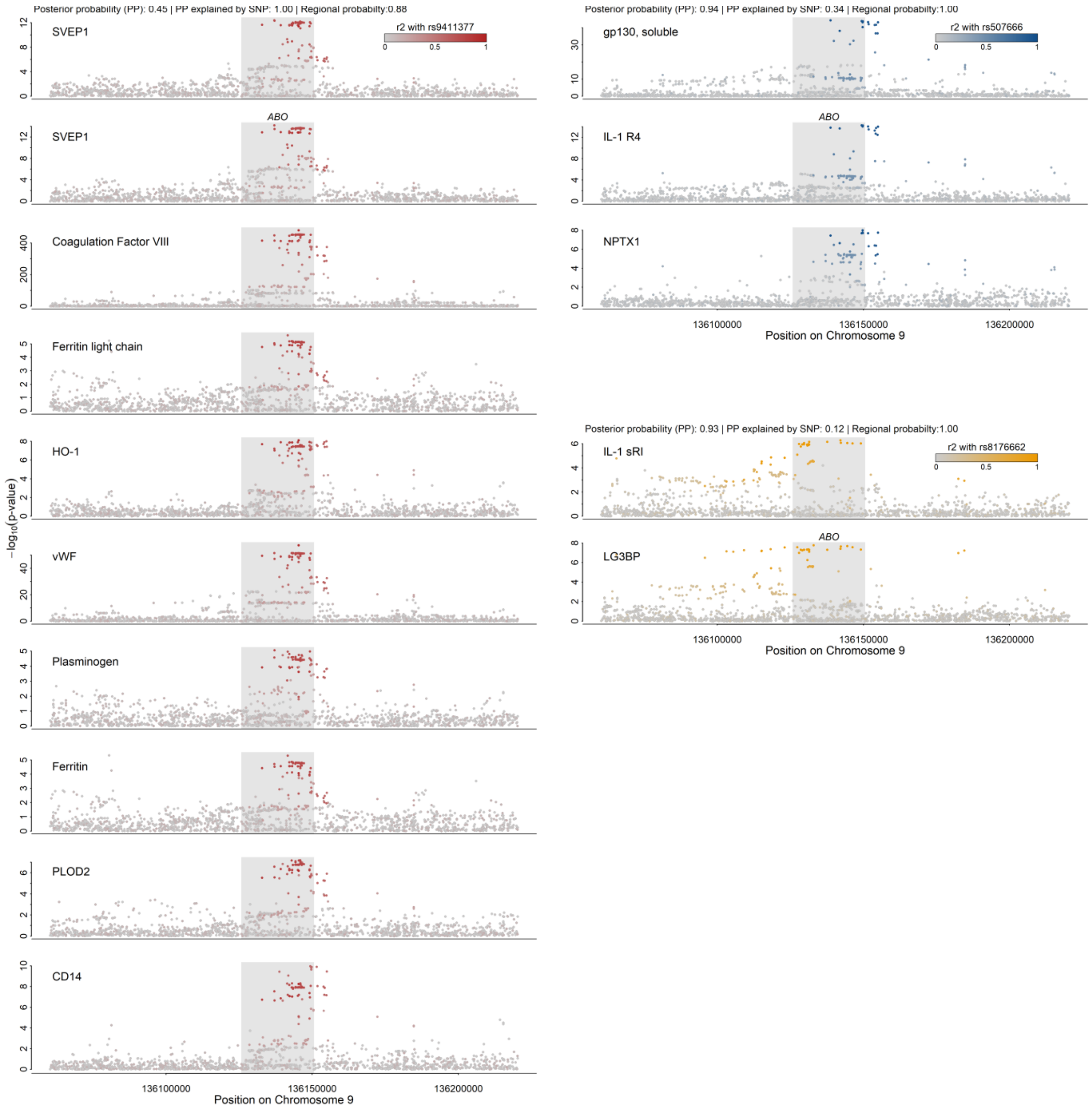

Figure S5 Regional association plots centred on the *ABO* locus. Multi-trait colocalization identified three cluster (red, blue, and orange) of protein associations distributed across three distinct likely causal variants. P-values are coloured based on linkage disequilibrium (r2) with the candidate variant prioritized by the colocalization algorithm (red, blue, and orange). Probabilities are given on top of each cluster. Protein names are shown in the upper right corner of each plot.
