# Supplementary material for: Genetic architecture of host proteins involved in SARS-CoV-2 infection
Source: Nat Commun. 2020 Dec 16;11:6397. doi: 10.1038/s41467-020-19996-z (PMC7744536; doi:10.1038/s41467-020-19996-z)
Supplement: Supplementary file 3 — Description of Additional Supplementary Files [file 41467_2020_19996_MOESM3_ESM.pdf]

Description of Additional Supplementary Files

Supplementary Data 1
Summary of SARS-CoV-2 or COVID-19 related proteins.

Supplementary Data 2
Summary of high-priority SOMAmers, i.e. with at least one cis-pQTL

Supplementary Data 3
Regional sentinel pQTLs

Supplementary Data 4
Results from conditional analysis

Supplementary Data 5
Comparison of effect estimates between SOMAscan and Olink assays

Supplementary Data 6
Significant results from SOMAmer cis-score analyses in UK Biobank.

Supplementary Data 7
Look-up of 220 cis-pQTLs and proxies in LD ($r2>0.8$) in various data bases
