# Supplementary material for: Genetic architecture of host proteins involved in SARS-CoV-2 infection
Source: Nat Commun. 2020 Dec 16;11:6397. doi: 10.1038/s41467-020-19996-z (PMC7744536; doi:10.1038/s41467-020-19996-z)
Supplement: Supplementary file 2 — Peer Review File [file 41467_2020_19996_MOESM2_ESM.pdf]

Reviewer #1 (Remarks to the Author):

Key Results

This work takes advantage of an existing genotyping population-based cohort (The Fenland Study) of > 12,000 individuals with genotype array and phenotypic data available, and harnesses this to look for genetic evidence linking candidate COVID related proteins to molecular and clinical phenotypes.
Of 179 proteins measured using an apatamer based method (Somascan), in plasma from 10,708 individuals, 220 cis pQTLs were identified for 97 proteins. For some of these proteins, genetic factors explained the majority of explained variance, suggesting these may be biologically meaningful effects, at least in health. For 45 proteins these cis pQTLS are the first reported. Hypothesis generating links with clinical disease are drawn: In some cases the variants could be linked to biobank clinical outcomes, for example a thrombin genetic risk score associated with increased risk of embolism, a major complication in COVID disease, and a specific focus on two COVID GWAS signals highlighted 10 proteins sharing a genetic signal in linkage with the ABO locus, suggesting possible linked pathways involved in regulation of coagulation.

Validity
The paper depends heavily on the aptamer based protein assay. Less than half the candidate COVID related proteins (179 of a possible 409 44%) were targetable with the assay, limiting the scope of the study, which eventually focuses on only 97 proteins with pQTLs. The authors acknowledge in addition that some variants may alter binding and thus bias results. A subset of estimates were cross validated with the O-link assay and reassuringly good correlation was found. The authors suggest that the polyclonal nature of the O-link antibodies reduces the effect of protein variants on binding, but do not justify why Somascan was chosen for the bulk of the analysis in this case (2 authors are Somascan employees).
A further caution is that the protein levels are entirely in plasma, this is particularly an issue when comparisons are made with tissue specific GTEX expression data. Is protein level tissue specific data, available in the human protein atlas, consistent with the population level GTEX expression data?
The selection of putative COVID proteins is a rather mixed bag, perhaps unavoidably given the novelty of the disease and preliminary nature of most sources. The interaction partners are of most interest since there is a possible biological rationale. Many of the markers of disease severity and adverse prognosis are very non specific markers of severe disease / sepsis and less likely to be primary or specific drivers of disease due to COVID.
A major limitation of the study, since it is built on an existing database of healthy individuals recruited pre-COVID, is that it cannot tell us whether the genetic – proteomic -phenotypic links detected either persist, or have any relevance, in the context of SARS-CoV-2 infection. It would have been good to see some comparisons made with data from emerging COVID specific human genomic databases, such as the COVID-19 host genetics initiative.
Notably the fenland cohort excluded diabetics or 'inability to walk unaided' thus excluding many of the people most vunerable to severe disease with SARS-CoV-2 infection. Ethnicity data for the Fenland participants should be summarised in the methods.
Hypothetically key cis pQTLS from this study might be enriched in patients with specific outcomes (e.g. thrombosis and the thrombin-cis-GRS).
Some of the findings would clearly be strengthened by additional validation in vitro or in vivo models. For example the MARK3 variant would be interesting to explore in a hACE2 murine model, however this is beyond the scope of the current paper.

Originality
The most original element here is the novel genetic associations with proteins which are likely to be the focus of much current research interest. Supportive evidence for novel SARS-CoV-2 therapeutic targets is clearly urgently needed and this paper provides hypothesis generating data to motivate further validation work in this area.
The drug target analysis is less original, since the proteins discussed are from existing databases.

However, a number of these drugs are already in clinical trials for COVID-19 (e.g. toculizimab, eculizimab) and the genetic variant effects highlighted here could be useful for stratifying trial participants or mendelian randomisation.
While data from the Fenland cohort has previously been published, this targeted proteomic analysis for COVID-19 is new and timely, though not a novel method as the authors have applied this to develop protein driven biological models for a range of other diseases.

Data and Methods
Detailed and clear methods are supplied, and the tables allow easy trawling for favourite proteins. The associated website provides raw data and analysis for comprehensive study and replication. I may be wrong but I think the reference in line 96-97 to 22 proteins highlighted by Gordon et al, ref 3, is incorrect – 22 appears to be the number of proteomic markers from reference 7.
The statistical methods appear appropriate, though I cannot comment expertly on the colocalization analysis.

Reviewer #2 (Remarks to the Author):

Genetic architecture of host proteins interacting with SARS-CoV-2

Langenberg and team have undertaken an in silico assessment of pQTLs for 179 host proteins that are thought to interact with SARS-CoV-2 proteins, or the host response. They report a link between MARK3 and variation at the ABO locus.

This is an excellent manuscript, timely and helpful to the field. They should be congratulated for getting this done so quickly and with rigour. The webserver is very useful.

I do have a few comments, which I hope may improve the manuscript.

Major comments
1) Overall, the manuscript presents highly relevant data for cis-pQTLs from an excellent sample, which is large and well-characterized. However, in general, the paper is more a description of a set of tables, rather than a narrative. It would help the reader to follow a story throughout the manuscript, such as the MARK3 locus.
2) Is there any information on COVID status on the Fenland individuals. Obviously this would be helpful.
3) The blood group O results from the NEJM Hostage paper could very well be spurious. This is because the control group was often blood donors. Blood donors are often group O and this may have confounded their results.
4) The authors need to describe in their results how they differentiated between horizontal and vertical pleiotropy.
5) The importance of the genetic correlation matrix is not clear to me. Please remind us how this is relevant to the problem at hand.
6) The authors should consider colocalization limitations, given the assumptions of these methods and describe these limitations.
7) Some feedback on the website:
a. It's great!
b. However, when looking at the GWAS statistics, it would be helpful to provide more detail. When you state "Target" does that mean the effect of the effect allele on the level of the "Target"?
c. Don't write A2 and A1. Use Effect allele and non-effect allele please!!
d. What is the effect in units?
e. Describe the meta-analysis
f. I queried IFNB1 and got a list of targets that do not contain IFNB1. Why is that?

Minor
1) The references are poorly organized. For reference 7, I could not find the article.
1) Why was an FDR of 10% used for the association of cis-GRS for ICD outcomes in UKB?

Reviewer #3 (Remarks to the Author):

This timely paper describes an important study of proteins related to a novel coronavirus currently ravaging the entire planet.
The genetic influence on protein abundance of 179 human proteins potentially-related to COVID-19 infection or disease severity may help us understand individual susceptibility and disease progression in infected individuals.

While overall the paper is very good and thorough, there are two areas I want to draw attention to:

1) The ABO locus. Across all GWAS, this is one of the most highly pleiotropic loci known, for a variety of direct and indirect biological reasons. In particular, however, the most recent results from the covid19 host genetics initiative show no association with covid19 infection and the ABO locus. The authors should carefully review these results and possibly remove the discussion of ABO from throughout the manuscript.

2) As the authors note, most drug targets are proteins and also there are numerous mechanisms to influence protein abundance beyond gene expression. I encourage the authors to consider rewriting the eQTL section. For example, If I'm interpreting figure 5 correctly, at almost every locus PrediXcan prioritized a gene other than the gene encoding the protein recognized by the aptamer.

I don't like to recommend wording, but this example illustrates a reasonable interpretation of the results:

a. "Plasma levels of proteins depend on multiple biological processes, including expression of the encoding genes. Although eQTL signals can be highly pleiotropic, we sought to identify which cis-pQTLs could be explained by cis-eQTLs. We applied PrediXcan to generate predicted gene expression across the loci which revealed multiple significant gene expression models at each locus, with the most significant model being the protein coding gene (15? I didn't calculate)% of the time. Nevertheless, at 65 loci we did see significant association between predicted gene expression of the protein coding gene and protein abundance"

Other scientific comments

I assume all samples were collected prior to the covid-19 pandemic. I guess it's obvious but it might be worth stating that the protein levels and genetic associations do not reflect responses to infection with this novel coronavirus.

Definition of cis-pQTL (500 kb?) should be stated explicitly in the text. And is it from TSS or the full gene body?

In addition to genetic correlation among proteins it would be nice to know the observational correlations. This could fill the top half of Figure 3

On line 132: horizontal and vertical pleiotropy for cis-pQTLs. I had trouble interpreting this. If not essential, it could be omitted, or referenced in the context of the trans-pQTLs, or in the description of the GWAS catalog look-ups.

On line 166: I very much like the tiered system for the trans-pQTLs. It might be a bit much to say "in the absence of an accepted gold standard' and just say "for this analysis we introduced the following pragmatic…"
I followed the examples of horizontal pleiotropy. Is there also an example of vertical pleiotropy in the trans-pQTLs?

Line 178: typo in rsid: rs4648046 is on chromosome 4, not chromosome 1
The authors may mean rs4658046 instead. All rsids mentioned in the text should be double checked

Line 256: Sorry – I can't parse this sentence: "The higher plasma levels among individuals with genetically higher BMI and lower kidney function, however, do not reflect the fact that both of these are considered to be risk factors for COVID-19." I'm not sure what point that sentence is trying to make.

If I've correctly interpreted the analysis discussed at line 271, it sounds like a gene set enrichment of the predixcan-identified genes. But if most of these are false positives, what do we learn from this enrichment?

The section on drugability should make a stronger case for how the genetic information contributes to our understanding. Most of the information presented starting at line 320 could have been written without any of the results generated in this study.
The last sentence of the whole section hints at the value of the genetic information ("The cis-pQTLs we identified for PGES2 might be useful to explore this further"). This concept could be expanded and presented at the top of this section.
I assume Finan et al was the source of the drugability information in this section but I don't think there's a citation to it in this section.

Other stylistic recommendations.

In general the writing is very clear. However I found myself getting lost within and between paragraphs. I think the paper would be stronger by ensuring that each paragraph starts with a topic sentence that foreshadows the contents of that paragraph, and ensuring that each new idea earns a paragraph break.

For example, the paragraph starting at line 175 is all about the horizontal pleiotropy. The intro sentence should reflect this.
The descriptive stats of distribution can be tacked on to the end of the previous paragraph

As another example, the mention of SCALLOP (line 316) could start a new paragraph:

On line 88, it would be clearer to state explicitly: "We identified candidate COVID19-relevant proteins"

Figure 2 (manhattan) while traditional could be omitted. I prefer the 2 dimensional cis- and trans-pQTL plot as in Sun et al.

Figure 6 is also very pretty but it's difficult to pull useful information out of it.
I would prefer to see that figure omitted and possibly replaced with a screen shot from the web server which I find to be highly useful.

Overall I find this to be a highly relevant and timely contribution.

REVIEWER COMMENTS

Reviewer #1 (Remarks to the Author):

Key Results

This work takes advantage of an existing genotyping population-based cohort (The Fenland Study) of > 12,000 individuals with genotype array and phenotypic data available, and harnesses this to look for genetic evidence linking candidate COVID related proteins to molecular and clinical phenotypes.

Of 179 proteins measured using an apatamer based method (Somascan), in plasma from 10,708 individuals, 220 cis pQTLs were identified for 97 proteins. For some of these proteins, genetic factors explained the majority of explained variance, suggesting these may be biologically meaningful effects, at least in health. For 45 proteins these cis pQTLS are the first reported. Hypothesis generating links with clinical disease are drawn: In some cases the variants could be linked to biobank clinical outcomes, for example a thrombin genetic risk score associated with increased risk of embolism, a major complication in COVID disease, and a specific focus on two COVID GWAS signals highlighted 10 proteins sharing a genetic signal in linkage with the ABO locus, suggesting possible linked pathways involved in regulation of coagulation.

Validity

The paper depends heavily on the aptamer based protein assay. Less than half the candidate COVID related proteins (179 of a possible 409 44%) were targetable with the assay, limiting the scope of the study, which eventually focuses on only 97 proteins with pQTLs. The authors acknowledge in addition that some variants may alter binding and thus bias results. A subset of estimates were cross validated with the O-link assay and reassuringly good correlation was found. The authors suggest that the polyclonal nature of the O-link antibodies reduces the effect of protein variants on binding, but do not justify why Somascan was chosen for the bulk of the analysis in this case (2 authors are Somascan employees).

Thank you for the suggestion, we now emphasize the details of our strategy and much broader proteomic coverage of the SOMAscan technology more clearly (p4, line 92). Mapping of COVID-related proteins to the SOMAscan v4 assay was a pragmatic choice since it utilises large-scale population data from the most comprehensive proteomic assay currently available (5,000 human protein targets). Across 12 Olink panels 1,069 unique proteins are covered, presenting the entirety of available assays from Olink at the time of our study, only 32 have been shown to be relevant for either SARS-CoV-2 or COVID-19.

A further caution is that the protein levels are entirely in plasma, this is particularly an issue when comparisons are made with tissue specific GTEX expression data. Is protein level tissue specific data, available in the human protein atlas, consistent with the population level GTEX expression data?

We agree that measurement of bioactive molecules, such as proteins or transcripts, from blood samples comes with several challenges in terms of the biological relevance in specific tissues of interest. We utilized GTEx data to test whether identified pQTLs are also eQTLs and hence the altered abundance in plasma might be best explained by changes in differential expression of protein encoding transcripts in target tissues of high relevance to SARS-CoV-2/COVID-19.

Recent work from the GTEx consortium (Jiang et al. 2019 BioRxiv) revealed a broad and tissue-specific range of correlation coefficients between gene and protein expression, and poor correlation was in part attributed to the secretion of proteins into plasma, form organs such as the liver. We can only speculate on the exact tissue origin of plasma protein abundances among our study samples but linkage based on common genetic variation is a powerful tool to integrate diverse data sets.

The selection of putative COVID proteins is a rather mixed bag, perhaps unavoidably given the novelty of the disease and preliminary nature of most sources. The interaction partners are of most interest since there is a possible biological rationale. Many of the markers of disease severity and adverse prognosis are very non-specific markers of severe disease / sepsis and less likely to be primary or specific drivers of disease due to COVID.

Yes, we completely agree. Our aim was to be most inclusive for potential druggable targets related to the SARS-CoV-2 and its associated disease COVID-19, and to clearly distinguish the prior rationale for inclusion for each protein. We agree that the interaction partners are of substantial interest but it is also clear that the host inflammatory response causally contributes to disease pathogenesis, as recently emphasized by the successful repurposing of dexamethasone, a corticosteroid suppressing the hyperimmune response of the host, reducing mortality among COVID-19 patients requiring mechanical ventilation (Horby et al. 2020, MedRxiv). Because individual components of the inflammatory response e.g. interleukin-6 and other cytokines can be targeted directly, we included those that have been shown to be elevated in patients with COVID-19. Venous and arterial thrombosis has also been shown to be central to disease pathogenesis and there are now multiple trials of different anti-coagulation strategies underway so it is reasonable to consider coagulation proteins as potential therapeutic targets.

A major limitation of the study, since it is built on an existing database of healthy individuals recruited pre-COVID, is that it cannot tell us whether the genetic – proteomic -phenotypic links detected either persist, or have any relevance, in the context of SARS-CoV-2 infection. It would have been good to see some comparisons made with data from emerging COVID specific human genomic databases, such as the COVID-19 host genetics initiative.

Notably the fenland cohort excluded diabetics or ‘inability to walk unaided’ thus excluding many of the people most vunerable to severe disease with SARS-CoV-2 infection. Ethnicity data for the Fenland participants should be summarised in the methods.

The aim of our study and its design was to rapidly provide results for exactly this purpose, in independent samples and at a time that GWAS case numbers were sufficiently large to allow robust testing of whether genetic susceptibility to SARS-CoV-2 infection or COVID-19 prognosis are associated with genetic variation demonstrated to affect levels of specific host proteins in the general population. For this purpose, genetic instruments have to be specific for the protein of interest, i.e. minimizing confounding from other sources in particular severe diseases, and have to be derived in independent cohorts to avoid biased inference due to reverse causality. We exemplified the value of

our resource using the results of the so far only peer-reviewed GWAS on COVID-19 and highlight the dependence of several coagulation factors on blood group types or activity of the respective glycosyltransferase.

We added the information on ethnicity to the study description (p18, lines 457).

Hypothetically key cis pQTLS from this study might be enriched in patients with specific outcomes (e.g. thrombosis and the thrombin-cis-GRS).

Yes, thank you, we have now clarified this better. This is indeed one of the advantages of performing genetic analyses of protein levels in apparently healthy individuals and before disease onset, to clearly identify pathways leading to diseases. We used results from independent studies, selected to represent the largest available data sets, including case-control GWAS for different diseases, to test for phenotypic consequences of the identified pQTLs.

Some of the findings would clearly be strengthened by additional validation in vitro or in vivo models. For example the MARK3 variant would be interesting to explore in a hACE2 murine model, however this is beyond the scope of the current paper.

Thank you, this would obviously be a great follow-up of our hypothesis-generating study, but, as the reviewer correctly states, is beyond the scope of our work.

Originality

The most original element here is the novel genetic associations with proteins which are likely to be the focus of much current research interest. Supportive evidence for novel SARS-CoV-2 therapeutic targets is clearly urgently needed and this paper provides hypothesis generating data to motivate further validation work in this area.

The drug target analysis is less original, since the proteins discussed are from existing databases. However, a number of these drugs are already in clinical trials for COVID-19 (e.g. toculizimab, eculizimab) and the genetic variant effects highlighted here could be useful for stratifying trial participants or mendelian randomisation.

Repurposing of existing drugs clearly represents the most efficient way to identify treatments for COVID-19 so we see the inclusion of already-drugged proteins as a strength. We did further incorporate information on putative druggable targets based on specific properties of proteins using in-silico prediction as outlined in Finan et al. 2017., but these findings have less immediate prospect for translation into treatments for ongoing pandemic.

While data from the Fenland cohort has previously been published, this targeted proteomic analysis for COVID-19 is new and timely, though not a novel method as the authors have applied this to develop protein driven biological models for a range of other diseases.

We thank the reviewer for highlighting the particular novelty of our study and note that our previous work on predictive modelling is distinct to the identification of genetic determinates of circulating proteins.

Data and Methods

Detailed and clear methods are supplied, and the tables allow easy trawling for favourite proteins. The associated website provides raw data and analysis for comprehensive study and replication. I may be wrong but I think the reference in line 96-97 to 22 proteins highlighted by Gordon et al, ref 3, is incorrect – 22 appears to be the number of proteomic markers from reference 7.

We clarified the statement, since the 22 referred to druggable targets identified by Gordon et al. in addition to what Finan et al. proposed (p4, line 98).

The statistical methods appear appropriate, though I cannot comment expertly on the colocalization analysis.

Reviewer #2 (Remarks to the Author):

Genetic architecture of host proteins interacting with SARS-CoV-2

Langenberg and team have undertaken an in silico assessment of pQTLs for 179 host proteins that are thought to interact with SARS-CoV-2 proteins, or the host response. They report a link between MARK3 and variation at the ABO locus.

This is an excellent manuscript, timely and helpful to the field. They should be congratulated for getting this done so quickly and with rigour. The webserver is very useful.

We are delighted about the enthusiasm of the reviewer and thank you for this supportive statement.

I do have a few comments, which I hope may improve the manuscript.

Major comments

1) Overall, the manuscript presents highly relevant data for cis-pQTLs from an excellent sample, which is large and well-characterized. However, in general, the paper is more a description of a set of tables, rather than a narrative. It would help the reader to follow a story throughout the manuscript, such as the MARK3 locus.

Thank you, we have now revised several sections in the manuscript to demonstrate the utility and insights from the core analyses of our work using examples, as suggested (e.g. p13, lines 342-347). We appreciate that the reviewer understands that it is a difficult balance to strike to rapidly provide a resource that systematically provides as much detail as possible, enables others to use and understand the results and tools as well as possible, while focussing sufficiently on key results to provide an engaging narrative.

2) Is there any information on COVID status on the Fenland individuals. Obviously this would be helpful.

In line with other UK and international cohorts, we are currently in the process of setting up a third phase that will follow-up on COVID-19 related outcomes among Fenland participants. This information will unfortunately not be available any time soon.

3) The blood group O results from the NEJM Hostage paper could very well be spurious. This is because the control group was often blood donors. Blood donors are often group O and this may have confounded their results.

We agree that the ABO locus should be treated with caution, as also pointed out by reviewer 3. We have carefully revised the corresponding section and deleted any possible overstating of this finding and added recent concerns to the discussion (p16, lines 424-433). We would, however, prefer to keep the ABO as an example to show a possible application of our findings, since blood group status has been associated with COVID-19 outcomes in several observational studies (see revised manuscript p16, lines 424-426) and different variants at the ABO locus are consistently associated with multiple proteins with relevance to COVID-19.

4) The authors need to describe in their results how they differentiated between horizontal and vertical pleiotropy.

We revised our definition of horizontal and vertical pleiotropy of pQTLs to indicate more clearly which pattern of associations we attribute to vertical and which to horizontal pleiotropy (p5, lines 119-127).

5) The importance of the genetic correlation matrix is not clear to me. Please remind us how this is relevant to the problem at hand.

A fundamental property of proteins is the ability to interact with each other and the genetic correlation matrix can help identify potential interaction partners, including such between putative SARS-CoV-2 interaction partners and proteins related to the maladaptive response of the host. It further helps to fill gaps in our knowledge about the relation among those proteins relevant to SARS-CoV-2/COVID-19, since apart from cellular models not much is known about the relevance of those proteins in the circulation at all. In other words, while we motivate the study to help facilitate identification of drug targets for COVID-19 we also wanted to gain insights in so far poorly characterized host proteins.

6) The authors should consider colocalization limitations, given the assumptions of these methods and describe these limitations.

We extended our previous concerns about colocalization, in particular the one causal variant assumption, in the revised version of the manuscript (p17, lines 451-452).

7) Some feedback on the website:

a. It's great!

Thank you, we appreciate the enthusiasm of the reviewer.

b. However, when looking at the GWAS statistics, it would be helpful to provide more detail. When you state "Target" does that mean the effect of the effect allele on the level of the "Target"?

We thank the reviewer for this helpful advice and added additional explanations to the webserver, including a brief descriptions of terms used.

c. Don't write A2 and A1. Use Effect allele and non-effect allele please!!

We changed the column headers to clearly indicate the effect and the other allele.

d. What is the effect in units?

Effects are presented as increase/decrease in 1 SD unit of inverse-rank normalized and covariate-adjusted plasma aptamer abundances. We added this information to the webserver.

e. Describe the meta-analysis

We added the information about the meta-analysis to the webserver.

f. I queried IFNB1 and got a list of targets that do not contain IFNB1. Why is that?

Interferon beta was not included in our candidate list, since evidence for its relevance to COVID-19 has emerged only very recently, and apologies if there were unexpected results using the webserver. We are in the process of finalising the genetic discovery of the entire SOMAscan v4 assay, including interferon beta, and will make pQTLs for interrogation with COVID-19 GWAS available soon.

Minor

1) The references are poorly organized. For reference 7, I could not find the article.

We apologize for the misleading reference and have now corrected the paper and revised all other references.

2) Why was an FDR of 10% used for the association of cis-GRS for ICD outcomes in UKB?

We used an FDR of 10% to be more inclusive given the high sensitivity of the topic, i.e. we didn't want to miss any possible relevant outcomes, in particular given the low number of cases for some outcomes in UK Biobank.

Reviewer #3 (Remarks to the Author):

This timely paper describes an important study of proteins related to a novel coronavirus currently ravaging the entire planet.

The genetic influence on protein abundance of 179 human proteins potentially-related to COVID-19 infection or disease severity may help us understand individual susceptibility and disease progression in infected individuals.

While overall the paper is very good and thorough, there are two areas I want to draw attention to:

1) The ABO locus. Across all GWAS, this is one of the most highly pleiotropic loci known, for a variety of direct and indirect biological reasons. In particular, however, the most recent results from the covid19 host genetics initiative show no association with covid19 infection and the ABO locus. The authors should carefully review these results and possibly remove the discussion of ABO from throughout the manuscript.

We appreciate that the ABO locus might be a false positive signal given the possible biased selection of control individuals. We thoroughly revised all references to the ABO locus in the entire paper and deleted this section from the abstract (p16, lines 424-433). We did however keep some discussion on the ABO locus for two reasons: 1) several distinct variants at the ABO locus seem to be relevant for distinct proteins, which is of general interest for the use of pQTLs even beyond COVID-19, and 2) several observational studies have associated blood group status with severity of COVID-19 (p16, lines 424-426) and better powered GWAS studies, in particular for the need of hospitalisation among COVID-19 patients, may well identify the ABO locus as relevant.

2) As the authors note, most drug targets are proteins and also there are numerous mechanisms to influence protein abundance beyond gene expression. I encourage the authors to consider rewriting the eQTL section. For example, If I'm interpreting figure 5 correctly, at almost every locus PrediXcan prioritized a gene other than the gene encoding the protein recognized by the aptamer.

I don't like to recommend wording, but this example illustrates a reasonable interpretation of the results:

a. "Plasma levels of proteins depend on multiple biological processes, including expression of the encoding genes. Although eQTL signals can be highly pleiotropic, we sought to identify which cis-pQTLs could be explained by cis-eQTLs. We applied PrediXcan to generate predicted gene expression across the loci which revealed multiple significant gene expression models at each locus, with the most significant model being the protein coding gene (15? I didn't calculate)% of the time. Nevertheless, at 65 loci we did see significant association between predicted gene expression of the protein coding gene and protein abundance"

We followed this very helpful suggestion of the reviewer and revised the eQTL section to highlight the potential contribution of genes other than the protein encoding ones to plasma protein levels (p9, lines 234-242).

Other scientific comments

I assume all samples were collected prior to the covid-19 pandemic. I guess it's obvious but it might be worth stating that the protein levels and genetic associations do not reflect responses to infection with this novel coronavirus.

We included this helpful suggestion upfront in the description of the study population (p4, lines 76-77).

Definition of cis-pQTL (500 kb?) should be stated explicitly in the text. And is it from TSS or the full gene body?

We added the definition of cis-pQTLs to the main text, which refers to a 500kb window around the full gene body (p4, lines 80-81).

In addition to genetic correlation among proteins it would be nice to know the observational correlations. This could fill the top half of Figure 3

We added the observational correlation of proteins to the upper half of Figure 3.

On line 132: horizontal and vertical pleiotropy for cis-pQTLs. I had trouble interpreting this. If not essential, it could be omitted, or referenced in the context of the trans-pQTLs, or in the description of the GWAS catalog look-ups.

We rephrased the corresponding section for clarification (p5, lines 119-123).

On line 166: I very much like the tiered system for the trans-pQTLs. It might be a bit much to say "in the absence of an accepted gold standard' and just say "for this analysis we introduced the following pragmatic…"

We changed the introduction of this section accordingly.

I followed the examples of horizontal pleiotropy. Is there also an example of vertical pleiotropy in the trans-pQTLs?

We identified few examples with vertical pleiotropy, including a cis-pQTL (rs2289252) for coagulation factor XI specifically associated with 4 other members of the coagulation cascade (kinigon 1, alpha-2-macroglobulin, kallikrein B, plasma (Fletcher factor) 1, and thrombin). We added this example to the manuscript (p7, lines 182-185).

Line 178: typo in rsid: rs4648046 is on chromosome 4, not chromosome 1

The authors may mean rs4658046 instead. All rsids mentioned in the text should be double checked

We thank the reviewer for pointing out this important typo and revised all rsIDs throughout the manuscript.

Line 256: Sorry – I can't parse this sentence: "The higher plasma levels among individuals with genetically higher BMI and lower kidney function, however, do not reflect the fact that both of these are considered to be risk factors for COVID-19." I'm not sure what point that sentence is trying to make.

We rephrased the corresponding section to highlight the contradiction in our findings, i.e. those individuals with higher genetic susceptibility to established risk factors for COVID-19 tend to have lower plasma levels of a protein shown to relate to the severity of COVID-19 (p9, lines 225-232).

If I've correctly interpreted the analysis discussed at line 271, it sounds like a gene set enrichment of the predixcan-identified genes. But if most of these are false positives, what do we learn from this enrichment?

We rephrased the section to clearly indicate that gene set enrichment was based on likely true positive gene models, i.e. passing a stringent Bonferroni threshold of $p<10^{-6}$ (p10, lines 248-251).

The section on drugability should make a stronger case for how the genetic information contributes to our understanding. Most of the information presented starting at line 320 could have been written without any of the results generated in this study.

The last sentence of the whole section hints at the value of the genetic information ("The cis-pQTLs we identified for PGES2 might be useful to explore this further"). This concept could be expanded and presented at the top of this section.

We followed the helpful suggestion of the reviewer and revised the corresponding section now making a stronger link between our findings and the potential application to drug repurposing efforts for COVID-19 (p11, lines 287-300).

I assume Finan et al was the source of the drugability information in this section but I don't think there's a citation to it in this section.

We added the missing reference to this section.

Other stylistic recommendations.

In general the writing is very clear. However I found myself getting lost within and between paragraphs. I think the paper would be stronger by ensuring that each paragraph starts with a topic sentence that foreshadows the contents of that paragraph, and ensuring that each new idea earns a paragraph break.

For example, the paragraph starting at line 175 is all about the horizontal pleiotropy. The intro sentence should reflect this. The descriptive stats of distribution can be tacked on to the end of the previous paragraph

As another example, the mention of SCALLOP (line 316) could start a new paragraph:

On line 88, it would be clearer to state explicitly: "We identified candidate COVID19-relevant proteins"

We revised the entire paper to ensure a clear flow of ideas and concepts and implemented the stylistic suggestion provided by the reviewer.

Figure 2 (manhattan) while traditional could be omitted. I prefer the 2 dimensional cis- and trans-pQTL plot as in Sun et al.

We chose a traditional Manhattan plot over the cis/trans plot used in previous pQTLs studies to give an immediate overview of the most important findings of the study, i.e. those proteins we are most confident about for the proposed down-stream applications. We added a cis/trans plot to the Supplemental Figures, now Supplemental Figure S1.

Figure 6 is also very pretty but it's difficult to pull useful information out of it. I would prefer to see that figure omitted and possibly replaced with a screen shot from the web server which I find to be highly useful.

We hope that the reviewer can be persuaded to keep this figure in the main manuscript. It was designed to accompany the webserver to highlight three important aspects of our phenotypic follow-up in a single figure: 1) a rapid overview of the pleiotropy of identified pQTLs for each protein, i.e. colourful bars in the inner circle likely indicate unspecific effects, 2) a systematic comparison with results from colocalization analyses as opposed to simple variant look-ups, which is still the norm but has large potential to introduce bias, as shown, and c) identification of consistency of effects across phenotypes through integration of effect directions. We appreciate that in detail it is slightly hard to read, but the higher-level messages are obvious. Few studies provide such systematic evaluation and we hope that the figure provides context and encourages others to avoid presentation of 'selected' examples. In addition, we added a panel exemplifying the webserver as novel Figure 7.

Overall I find this to be a highly relevant and timely contribution.

Thank you for these kind words.

Reviewer #1 (Remarks to the Author):

Thank you for your detailed replies. I am happy that the all the points raised have been addressed fully, or otherwise where requested more detail has been provided in the text to clarify the nature of the dataset and methods. The paper now reads more clearly, enabling the reader to understand the novel and highly pertinent findings. The manuscript is in my view entirely suitable for publication in Ncomms.

Reviewer #2 (Remarks to the Author):

The authors have addressed my comments.

Reviewer #3 (Remarks to the Author):

I appreciate the extensive work the authors have done in responding to all the reviewers' comments including the addition of a heat-map of observed correlations of the protein levels.

I also appreciate the revisions to improve readability of the whole manuscript.

I do have one lingering concern related to what I perceive as an over-reliance on PrediXcan.

I understand the text has already been modified to address my previously stated concerns. But we still have a situation where the paper reports "a novel cis-pQTL for MARK3" (line 342), but then the PrediXcan results would say the MARK3 gene isn't the causal gene for MARK3 protein levels at the MARK3 locus; Figure 5 shows PrediXcan selects BAG5 as the causal gene in liver and ATP5MPL (C14orf2) as the causal gene in left ventricle. These genes also sit at the MARK3 locus and likely represent pleiotropy of the regulatory elements responsible for MARK3 expression.
If I correctly understand the enrichment analysis, BAG5 and ATP5MPL (C14orf2) would have been included, even though they are likely spurious false positive hits.

At a minimum I think the topic sentence should be modified to reflect greater uncertainty in PrediXcan's results:
E.g., change: "For the majority of protein targets PrediXcan revealed genes other than the protein-encoding gene as most strongly associated with pQTL data."
To: "For the majority of protein targets PrediXcan selected genes other than the protein-encoding gene as most strongly associated with pQTL data."

Minor suggestions/corrections:
Line 73: "an ubiquitously" should be "a ubiquitously"
Line 182: "We identified few variants" should be "We identified a few variants"
Line 184: "kinigon 1" should be "Kininogen 1"

REVIEWERS' COMMENTS

Reviewer #1 (Remarks to the Author):

Thank you for your detailed replies. I am happy that the all the points raised have been addressed fully, or otherwise where requested more detail has been provided in the text to clarify the nature of the dataset and methods. The paper now reads more clearly, enabling the reader to understand the novel and highly pertinent findings. The manuscript is in my view entirely suitable for publication in Ncomms.

We thank the reviewer for his/her helpful comments and are please to know that the revised version is now suggested to be published.

Reviewer #2 (Remarks to the Author):

The authors have addressed my comments.

We thank the reviewer for his/her helpful comments and are please to know that the revised version is now suggested to be published.

Reviewer #3 (Remarks to the Author):

I appreciate the extensive work the authors have done in responding to all the reviewers' comments including the addition of a heat-map of observed correlations of the protein levels.

I also appreciate the revisions to improve readability of the whole manuscript.

We are pleased to hear that our revisions improved the manuscript and are thankful for the helpful comments of the reviewer.

I do have one lingering concern related to what I perceive as an over-reliance on PrediXcan. I understand the text has already been modified to address my previously stated concerns. But we still have a situation where the paper reports "a novel cis-pQTL for MARK3" (line 342), but then the PrediXcan results would say the MARK3 gene isn't the causal gene for MARK3 protein levels at the MARK3 locus; Figure 5 shows PrediXcan selects BAG5 as the causal gene in liver and ATP5MPL (C14orf2) as the causal gene in left ventricle. These genes also sit at the MARK3 locus and likely represent pleiotropy of the regulatory elements responsible for MARK3 expression.

We agree with the reviewer that integration of GTEx data did not yielded the expected results for this and also other examples and the reported *cis*-pQTL for MARK3 may likely affect expression of other genes in close proximity as well. We note that, none of the genes reached the corrected statistical significance, i.e. accounting for all genes tested at this locus, in none blood tissues and that indeed in blood expression of *MARK3* was the strongest gene related to the protein product in the circulation, which aligns with enrichment of *MARK3* expression in eosinophils in the human protein atlas. However, more work is needed to integrate tissue expression data with circulating proteins.

If I correctly understand the enrichment analysis, BAG5 and ATP5MPL (C14orf2) would have been included, even though they are likely spurious false positive hits.

For enrichment analysis we considered only predicted gene expression passing a more stringent significance threshold to account for multiple testing which excluded those genes.

At a minimum I think the topic sentence should be modified to reflect greater uncertainty in PrediXcan's results:

E.g., change: "For the majority of protein targets PrediXcan revealed genes other than the protein-encoding gene as most strongly associated with pQTL data."

To: "For the majority of protein targets PrediXcan selected genes other than the protein-encoding gene as most strongly associated with pQTL data."

We thank the reviewer for thoroughly checking our analysis and followed this very reasonable suggestion of the reviewer and rephrased the sentence accordingly.

Minor suggestions/corrections:

Line 73: "an ubiquitously" should be "a ubiquitously"

Line 182: "We identified few variants" should be "We identified a few variants"

Line 184: "kinigon 1" should be "Kininogen 1"

We made the suggested corrections.
